# Ferroelectric Particles in Nematic Liquid Crystals with Soft Anchoring

**DOI:** 10.3390/molecules26041166

**Published:** 2021-02-22

**Authors:** Cristina Cirtoaje

**Affiliations:** Department of Physics, University Politehnica of Bucharest, Splaiul Independentei 313, 060042 Bucharest, Romania; cristina.cirtoaje@upb.ro

**Keywords:** freedericksz transition, ferroelectric naonoparticle

## Abstract

A theoretical evaluation of the electric Freedericksz transition threshold and saturation field is proposed for a liquid crystals composite with ferroelectric particles. Existing models consider a strong anchoring of nematic molecules on the glass support of the cell, but in this paper a soft molecular anchoring of molecules on the glass support and also on the ferroelectric nanoparticle’s surface is assumed. Thus, a finite saturation field was obtained in agreement with real systems. Calculations are made for planar configuration of positive dielectric anisotropy liquid crystals. The results are compared with data obtained on similar systems from different publications and the differences are discussed.

## 1. Introduction

The intense development of liquid crystal’s (LC) use in electronic display or other electro-optic applications, has led to a new research direction—nanoparticles dispersions in liquid crystals. There are many research papers studying the physical properties of various types of nanoparticles such as azo-dyes [1,2] quantum dots [3,4,5,6], ferromagnetic particles [7,8,9,10], carbon nanoparticles [11,12,13] and ferroelectric particles [14,15,16,17,18,19,20,21,22,23,24]. Over time, there have been several theoretical models proposed to describe the physical interactions between nanoparticles and nematic molecules and also their influences on the Freedericksz transition threshold [15,16,17]. The Freedericksz transition voltage and saturation field are important parameters when molecular behavior under external stimuli (fields or radiations) is discussed. Experiments were generally in good agreement with these models but in certain cases these parameters proved to be strongly dependent on the liquid crystal properties as well as on the particle’s surface. The properties have been discussed and as can be seen in [17], the anchoring configuration is different in a positive dielectric anisotropy host compared to one in which the host anisotropy is negative. The qualitative behavior was explained but the quantitative detailed characterization has not yet been presented. The anchoring energy on the substrate of the cell has great importance both for Freedericksz transition as well as for the saturation field. Generally, this energy is low compared to the free energy density of elastic forces or of the applied fields but, when we look towards reducing the power consumption of liquid crystal displays (LCDs) or to obtaining controllable devices, such as wave modulators, we have to take into account all the influences. Just as in the previously mentioned studies, this manuscript uses the elastic continuum theory to find the Freedericksz and saturation fields but without neglecting the anchoring strength. The results are theoretically simulated and compared to experimental results leading to an improved model that can be used by engineers developing electro-optical devices based on liquid crystal nanomaterials. As can be seen, the anchoring influence depends on the applied field, so, for a specific range of field intensity, it can be neglected and the previously developed method can be applied without a significant loss of information. There are also some situations where the influence is strong and, in these cases, the present model is more suitable.

## 2. Theory and Calculations

### 2.1. Theory and Calculations

**Figure 1:**
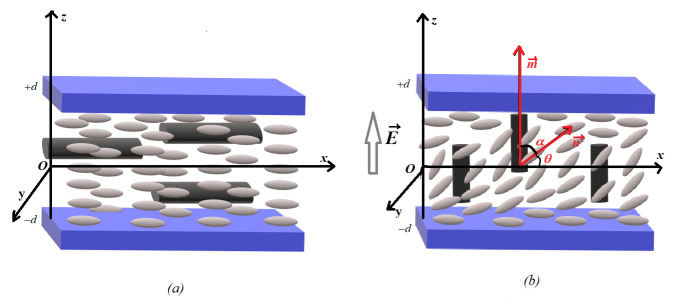
Molecular orientation in nematic liquid crystal with ferroparticles insertion (**a**) without an applied field and (**b**) with an electric field applied. The molecules and ferroparticles are oversized compared to the glass cell for a better understanding of orientation.

In planar aligned liquid crystals, the molecules are parallel to the support of the cell. If we consider cylindrical shape ferroelectric nanoparticles dispersed in liquid crystal, they will also present a specific orientation dictated by the physical and chemical properties of the host (Figure 1a). When an external stimulus (i.e., an electric field) is applied, the molecules change their orientation. This process is called the Freedericksz transition and it only appears when the field exceeds a threshold value (or a critical field). This field depends on many parameters, such as the dielectric properties of the host and those of dispersed particles, but also on the anchoring forces acting on the molecules around the nanoparticle’s surface.

Under the action of an electric field, these particles are expected to change their orientation more easily than do the liquid crystal molecules (Figure 1b). Depending on the anchoring strength, the overall orientation should be easier and a decrease in the Freedericksz transition threshold should be observed.

For the theoretical modeling of this system, the elastic continuum theory was used. This theory considers all the interactions between molecules—surface and inserted particles are elastic. The system’s stability is obtained when its free energy density is at a minimum (i.e., when it solves the Euler-Lagrange equation). First of all, let us determine the free energy. In the most simple manner, it can be written as:

(1) $$F=\int_{-d}^{+d}\left(f_{1}+f_{2}+f_{3}\right)dz+2F_{S}.$$

Here, $f_{1}$ is the elastic free energy density, $f_{2}$ is the interaction free energy density of the liquid crystal with the applied electric field, $f_{3}$ is the interaction free energy density of liquid crystal molecules and the ferroelectric nanoparticle’s surface. The $F_{S}$ term represents the anchoring energy on the substrate of the cell.

The first term of Equation (1) can be calculated according to elastic continuum theory as

(2) $$f_{1}=\frac{1}{2}K_{1}{\left(\nabla \vec{n}\right)}^{2}+\frac{1}{2}K_{2}{\left(\vec{n}\nabla \times \vec{n}\right)}^{2}+\frac{1}{2}K_{3}{\left[\vec{n}\times \left(\nabla \times \vec{n}\right)\right]}^{2},$$

where $K_{1}$ ,$K_{2}$ , $K_{3}$ are the splay, twist and bend elastic constants respectively and $\vec{n}$ is the nematic director. When the field is applied to the cell, the molecules change their orientation, making a new angle (θ) with the surface (Figure 1). Thus, Equation (2) becomes:

(3) $$f_{1}=\frac{1}{2}\left(K_{1}\cos^{2}\theta +K_{3}\sin^{2}\theta \right)\theta_{z}^{2},$$

where $\theta_{z}=\frac{d\theta }{dz}$.

The second term ($f_{2}$), representing the interaction free density energy between LC molecules and the applied field, is:

(4) $$f_{2}=-\frac{1}{2}\varepsilon_{0}\left(\varepsilon_{⊥}{\vec{E}}^{2}+\varepsilon_{a}{\left(\vec{n}\vec{E}\right)}^{2}\right).$$

Here, $\varepsilon_{0}$ represents the dielectric permittivity of vacuum, $\varepsilon_{⊥}$ is the perpendicular component of the composite dielectric permittivity and $\varepsilon_{a}$ is its dielectric anisotropy. By taking into account the deviation angle (θ) between the nematic director and the surface under the action of the applied electric field ($\vec{E})$, Equation (4) becomes

(5) $$f_{2}=-\frac{1}{2}\frac{D^{2}}{\varepsilon_{0}\left(\varepsilon_{⊥}+\varepsilon_{a}\sin^{2}\theta \right)},$$

where *D* is the dielectric displacement of the composite:

(6) $$D=\varepsilon_{0}E\left(\varepsilon_{⊥}+\varepsilon_{a}\sin^{2}\theta \right).$$

A simplified form can be obtained in terms of applied voltage *U* if we use the relationship between the applied voltage *U* and electric field intensity *E*:

(7) $$U=\int_{-d}^{d}E\left(z\right)dz.$$

Thus, we obtain

(8) $$f_{2}=-\frac{\varepsilon_{0}}{8d^{2}}\left(\varepsilon_{⊥}U^{2}+\varepsilon_{a}U^{2}\sin^{2}\theta \right).$$

Special attention must be paid to the molecular interaction with the inserted particles and with the alignment surface because they can strongly affect the Freedericksz transition and the saturation field.

For the interaction free energy density of nematic molecules with long cylindrical ferroelectic nanoparticles ($f_{3}$), the improved model of Zakhlevnikh and Burylov [25] is used. Thus, for a particle that has a cross-section diameter (*a*) and length (*l*), it can be written as:

(9) $$f_{3}=-A_{p}P_{2}\left(\cos\alpha \right){\left(\vec{m}\vec{n}\right)}^{2}\left(1-\xi_{p}{\left(\vec{m}\vec{n}\right)}^{2}\right).$$

Here, $A_{p}$ is a constant depending on anchoring energy (*W*), on volumetric fraction of ferroparticles (*f*) and on cross section diameter (*a*).

(10) $$A_{p}=\frac{2Wf}{a}.$$

As can be seen, the interaction energy density is not affected by the particle’s length (*l*), which is a good thing because this parameter is rarely the same for all the particles in the sample. The second term of Equation (9), $P_{2}\left(\cos\alpha \right)$, is the second Legendre polinomial:

(11) $$P_{2}\left(\cos\alpha \right)=\frac{3\cos^{2}\alpha -1}{2},$$

where α is the angle between the molecule and the ferroelectric particle’s surface illustrated in Figure 1b.

The vectors $\vec{n}$ and $\vec{m}$ are the molecular director and ferroparticle’s long axis, respectively. Finally, $\xi_{p}\;$ is a parameter for anchoring anisotropy on the ferroelectric particle’s surface. A detailed description of this parameter is given [26]. For $\xi_{p}=0,$ this interaction term presents a null minimum for a homeotropic alignment between molecule and ferroparticle ($\vec{m}⊥\vec{n}$). If 0 $\leq \xi_{p}\leq 0.5$, a decrease of the maximum point of interaction free energy density appears with the increase of $\xi_{p}.$ A second minimum, corresponding to a planar alignment of nematic molecules with the particle’s surface ($\vec{m}\;\left|\right|\;\vec{n}$), appears when $0.5<\xi_{p}\;<1$. When $\xi_{p}\;=1$, the interaction free energy density goes down to zero for ($\vec{m}⊥\vec{n}$) and for ($\vec{m}\;\left|\right|\;\vec{n}$).

This parameter is very important because, as can be seen from [17], the molecular orientation on the nanoparticle’s surface is crucial for the Freedericksz transition evolution with the increase of dopant concentration.

The first model of molecular interaction with the support surface was provided by Rapini and Papoular [27] as:

(12) $$F_{S}=\frac{1}{2}SA\sin^{2}\theta_{0},$$

where *A* is the anchoring strength, $\theta_{0}\to 0$ is is the angle between the direction of easy axe and nematic director and *S* is the contact surface between the nematic sample and the solid support. This model was improved by Guochen and collaborators [28] by considering the alignment angle of small perturbations:

(13) $$F_{S}=\frac{SA_{S}}{2}\sin^{2}\theta_{0}\left(1+\xi_{S}\sin^{2}\theta_{0}\right).$$

Here, ($\xi_{S}$) is a parameter depending on the surface properties and, as can be seen from Equation (12), if $\xi_{S}=0$, the same surface energy as the classical Rapini-Papoular model is obtained.

### 2.2. Freedericksz Transition Threshold

The Freedericksz transition mainly affects the molecules placed in the middle of the cell. These molecules are not attached to the surface so the interaction term with the support surface will not be considered here. Thus, the total free energy density inside the cell, denoted with $f_{T}$, will be taken into account :

(14) $$f_{T}=f_{1}+f_{2}+f_{3}.$$

When an external field is applied to the liquid crystal, a small distortion is induced in its structure. The stationary state of this new structure is obtained only when its free energy reaches its minimum, that is, when the Lagrange equation is satisfied by the deviation angle θ.

(15) $$\frac{d}{dz}\left(\frac{\partial f_{T}}{\partial \theta_{z}}\right)-\frac{\partial f_{T}}{\partial \theta }=0.$$

Because $f_{T}$ is independent of *z* coordinate from (15) we get:

(16) $$\theta_{z}\frac{\partial f_{T}}{\partial \theta_{z}}-f_{T}=C,$$

where *C* is a constant value.

Considering Equations (3), (7) and (9), in Equation (16) we get:

(17) $$\frac{1}{2}\left(K_{1}\cos^{2}\theta +K_{3}\sin^{2}\theta \right)\theta_{z}^{2}+\frac{\varepsilon_{0}}{8d^{2}}\left(\varepsilon_{⊥}U^{2}+\varepsilon_{a}U^{2}\sin^{2}\theta \right)+A_{p}\sin^{2}\theta \left(1-\xi_{p}\sin^{2}\theta \right)=C.$$

Since *C* is a constant, the equation above is an identity and it is true for any *z* value, including z=0, where $\theta_{z}=0$ and $\theta =\theta_{m}$ (the maximum deviation angle reached in the middle of the cell).

So, we get from Equations (17) and (18)

(18) $$C=\frac{\varepsilon_{0}}{8d^{2}}\left(\varepsilon_{⊥}U^{2}+\varepsilon_{a}U^{2}\sin^{2}\theta_{m}\right)+A_{p}\sin^{2}\theta_{m}\left(1-\xi_{p}\sin^{2}\theta_{m}\right)$$

and

(19) $$\frac{d\theta }{dz}=\sqrt{\frac{Z\left(\theta ,\theta_{m}\right)}{g\left(\theta \right)}},$$

where

(20) $$g\left(\theta \right)=\frac{1}{2}\left(K_{1}\cos^{2}\theta +K_{3}\sin^{2}\theta \right)$$

(21) $$\begin{array}{c} Z\left(\theta ,\theta_{m}\right)=\left(\frac{\varepsilon_{0}\varepsilon_{a}U^{2}}{8d^{2}}+A_{p}\right)\left(\sin^{2}\theta_{m}-\sin^{2}\theta \right)-A_{p}\xi_{p}\left(\sin^{4}\theta_{m}-\sin^{4}\theta \right). \end{array}$$

Here, we must take into account that $\theta =\theta_{0}$ for z=−d and in the middle of the cell where z=0, the distortion angle θ reaches its maximum value, $\theta_{m}$. Thus, by integrating Equation (19) we get:

(22) $$d=\int_{\theta_{0}}^{\theta_{m}}\sqrt{\frac{g\left(\theta \right)}{Z\left(\theta ,\theta_{m}\right)}}d\theta .$$

To simplify the calculations, we change the variable into λ, where

(23) $$\sin\lambda =\frac{\sin\theta }{\sin\theta_{m}}$$

and we get

(24) $$d\theta =\frac{\sqrt{1-\sin^{2}\lambda }\sin\theta_{m}}{\sqrt{1-\sin^{2}\lambda \sin^{2}\theta_{m}}}d\lambda .$$

Thus, after replacing the variables from Equations (20) to (24), we obtain for *d* the following form:

(25) $$d=\int_{\lambda_{0}}^{\frac{\pi }{2}}\sqrt{\frac{K_{1}+\left(K_{3}-K_{1}\right)\sin^{2}\theta_{m}\sin^{2}\lambda }{\left(1-\sin^{2}\theta_{m}\sin^{2}\lambda \right)Y\left(\theta ,\theta_{m}\right)}}d\lambda ,$$

where we used the simplified notation $Y\left(\lambda ,\theta_{m}\right)$ for:

(26) $$\begin{array}{c} Y\left(\lambda ,\theta_{m}\right)=\frac{\varepsilon_{0}\varepsilon_{a}U^{2}}{4d^{2}}+2A_{p}\left[1-\xi_{p}\sin^{2}\theta_{m}\left(1+\sin^{2}\lambda \right)\right]. \end{array}$$

The Freedericksz transition appears when the changing of the structure is at its beginning, so the maximum distortion angle (the distortion angle in the middle of the cell) is quite small and we can write $\theta_{m}\to 0$. When the distortions are so small, the system’s alignment is almost planar and we can write the electric displacement as:

(27) $$D=\varepsilon_{0}\varepsilon_{⊥}E$$

for the Fredericksz transition displacement $\left(D_{F}\right)$ and voltage $\left(D_{F}\right)$ it becomes:

(28) $$D_{F}=\varepsilon_{0}\varepsilon_{⊥}\frac{U_{F}}{2d}.$$

Thus we obtain for the cell thickness:

(29) $$d=\sqrt{\frac{K_{1}}{\frac{\varepsilon_{a}D_{F}^{2}}{\varepsilon_{0}\varepsilon_{⊥}}+2A_{p}}}\left(\frac{\pi }{2}-\lambda_{0}\right),$$

from which we get

(30) $$\frac{d}{\sqrt{K_{1}}}\sqrt{\frac{\varepsilon_{a}D_{F}^{2}}{\varepsilon_{0}\varepsilon_{⊥}^{2}}+2A_{p}}=\frac{\pi }{2}-\lambda_{0}.$$

By applying the cot function to Equation (30) we obtain:

(31) $$\cot\left(\frac{d}{\sqrt{K_{1}}}\sqrt{\frac{\varepsilon_{a}D_{F}^{2}}{\varepsilon_{0}\varepsilon_{⊥}^{2}}+2A_{p}}\right)=\tan\lambda_{0}.$$

The boundary conditions on the edges of the cell z=−d and z=d for the elastic interaction energy must be in agreement with the anchoring energy from Equation (2), which means that:

(32) $$\left(K_{1}\cos^{2}\theta_{0}+K_{3}\sin^{2}\theta_{0}\right){\left(\frac{d\theta }{dz}\right)}_{z=\pm d}=A_{s}\sin\theta_{0}\cos\theta_{0}\left(1+2\xi_{S}\sin^{2}\theta_{0}\right).$$

So we obtain:

(33) $${\left(\frac{d\theta }{dz}\right)}_{z=\pm d}=\frac{A_{s}\sin\theta_{0}\cos\theta_{0}\left(1+2\xi_{S}\sin^{2}\theta_{0}\right)}{\left(K_{1}\cos^{2}\theta_{0}+K_{3}\sin^{2}\theta_{0}\right)}.$$

We return to the previous notations:

(34) $$\sin\lambda_{0}=\frac{\sin\theta_{0}}{\sin\theta_{m}}$$

and we get:

(35) $${\left(\frac{d\theta }{dz}\right)}_{z=\pm d}=\frac{A_{S}\sin\theta_{m}\sin\lambda_{0}\sqrt{1-\sin^{2}\theta_{m}\sin^{2}\lambda_{0}}\left(1+2\xi_{S}\sin^{2}\theta_{m}\sin^{2}\lambda_{0}\right)}{K_{1}+\left(K_{3}-K_{1}\right)\sin^{2}\theta_{m}\sin^{2}\lambda_{0}}.$$

From Equation (19), it results that:

(36) $${\left(\frac{d\theta }{dz}\right)}_{z=\pm d}=\frac{\sin\theta_{m}\sqrt{\left(1-\sin^{2}\lambda_{0}\right)Y\left(\lambda_{0},\theta_{m}\right)}}{\sqrt{K_{1}+\left(K_{3}-K_{1}\right)\sin^{2}\theta_{m}\sin^{2}\lambda_{0}}},$$

where $Y\left(\lambda_{0},\theta_{m}\right)$ is given in Equation (26)

For $\theta_{m}\to 0$ in Equations (35) and (36) we get

(37) $$\tan\lambda_{0}=\frac{\sqrt{K_{1}}}{A_{s}}\sqrt{\frac{\varepsilon_{a}D_{F}^{2}}{\varepsilon_{0}\varepsilon_{⊥}^{2}}+2A_{p}}.$$

We finally obtain the transcendental equation from Equations (31) and (37):

(38) $$\cot\left(\frac{d}{\sqrt{K_{1}}}\sqrt{\frac{\varepsilon_{a}D_{F}^{2}}{\varepsilon_{0}\varepsilon_{⊥}^{2}}+2A_{p}}\right)=\frac{\sqrt{K_{1}}}{A_{s}}\sqrt{\frac{\varepsilon_{a}D_{F}^{2}}{\varepsilon_{0}\varepsilon_{⊥}^{2}}+2A_{p}}.$$

Equation (38) can be expressed as a function of Fredericksz transition threshold $U_{F}$ given in Equation (28),

(39) $$\cot\left(\frac{d}{\sqrt{K_{1}}}\sqrt{\frac{\varepsilon_{0}\varepsilon_{a}U_{F}^{2}}{4d^{2}}+2A_{p}}\right)=\frac{\sqrt{K_{1}}}{A_{s}}\sqrt{\frac{\varepsilon_{0}\varepsilon_{a}U_{F}^{2}}{4d^{2}}+2A_{p}}.$$

Equation (39) is a transcendental equation that can be numerically solved by replacing the constant parameters with their reported values in the literature. By giving different values to the $A_{S}$ parameter we can obtain simplified equations from which the Freedericksz voltage $U_{F}$ can be determined.

From the same equation we can also obtain the Freedericksz transition threshold for the nematic sample (without ferroparticle’s insertions) by considering f=0. Thus, we have $A_{p}=0$ and we denote the critical voltage with $U_{F0}$.

(40) $$\cot\left(\frac{d}{\sqrt{K_{1}}}\sqrt{\frac{\varepsilon_{0}\varepsilon_{a}U_{F0}^{2}}{4d^{2}}}\right)=\frac{\sqrt{K_{1}}}{A_{s}}\sqrt{\frac{\varepsilon_{0}\varepsilon_{a}U_{F0}^{2}}{4d^{2}}}.$$

### 2.3. Saturation Field

The saturation field is the critical value for which all the molecules are aligned by the field. That includes the molecules in the vicinity of the substrate so the boundary conditions from Equation (22) must be satisfied:

$$d=\int_{\theta_{0}}^{\theta_{m}}\sqrt{\frac{g\left(\theta \right)}{Z\left(\theta ,\theta_{m}\right)}}d\theta .$$

A change of variable is applied:

(41) $$\cos\lambda =\frac{\cos\theta_{m}}{\cos\theta }\;\;\;\cos\lambda_{0}=\frac{\cos\theta_{m}}{\cos\theta_{0}}.$$

Thus, we obtain:

(42) $$d\theta =-\frac{\cos\theta_{m}\sin\lambda }{\cos\lambda \sqrt{\cos^{2}\lambda -\cos^{2}\theta_{m}}}d\lambda$$

and

(43) $$d=\int_{0}^{\lambda_{0}}\sqrt{\frac{K_{1}+\left(K_{3}-K_{1}\right)\left(1-\frac{\cos^{2}\theta_{m}}{\cos^{2}\lambda }\right)}{Y_{1}\left(\lambda ,\theta_{m}\right)}}\frac{1}{\sqrt{\cos^{2}\lambda -\cos^{2}\theta_{m}}}d\lambda ,$$

where

(44) $$\begin{array}{c} Y_{1}\left(\lambda ,\theta_{m}\right)=\frac{\varepsilon_{0}\varepsilon aU^{2}}{4d^{2}}+2A_{p}\left\{1-\xi_{p}\left[2-\cos^{2}\theta_{m}\left(1+\frac{1}{\cos^{2}\lambda }\right)\right]\right\}. \end{array}$$

Saturation is reached when a complete reorientation is achieved, that is, $\theta_{m}\to \pi /2$ and the liquid crystal passes from planar to homeotropic alignment. In this case, $\cos\theta_{m}\to 0$, so we obtain:

(45) $$d=\int_{0}^{\lambda_{0}}\sqrt{\frac{K_{3}}{\frac{\varepsilon_{0}\varepsilon aU_{S}^{2}}{4d^{2}}+2A_{p}\left(1-2\xi_{p}\right)}}\frac{d\lambda }{\cos\lambda },$$

where $U_{S}$ is the saturation voltage, which can be expressed as a function of electric displacement considering a complete reorientation of molecules, that is,

(46) $$D_{S}=\varepsilon_{0}\varepsilon_{\|}E_{S}=\varepsilon_{0}\varepsilon_{\|}\frac{U_{S}}{2d}.$$

By integrating Equation (45) it results in

(47) $$d=\frac{1}{2}\sqrt{\frac{K_{3}}{\frac{\varepsilon_{0}\varepsilon aU_{S}^{2}}{4d^{2}}+2A_{p}\left(1-2\xi_{p}\right)}}\log\left(\frac{1+\sin\lambda_{0}}{1-\sin\lambda_{0}}\right),$$

from which we get:

(48) $$\sin\lambda_{0}=\tanh\left(\frac{d}{\sqrt{K_{3}}}\sqrt{\frac{\varepsilon_{a}\varepsilon_{0}U_{s}^{2}}{4d^{2}}+2A_{p}\left(1-2\xi_{p}\right)}\right).$$

Using the boundary conditions from Equation (32) and the variable change from Equation (41), we obtain:

(49) $${\left(\frac{d\theta }{dz}\right)}_{z=\pm d}=\frac{A_{S}\sqrt{1-\frac{\cos^{2}\theta_{m}}{\cos^{2}\lambda_{0}}}\left[1+2\xi_{S}\left(1-\frac{\cos^{2}\theta_{m}}{\cos^{2}\lambda_{0}}\right)\right]\frac{\cos\theta_{m}}{\cos\lambda_{0}}}{K_{1}+\left(K_{3}-K_{1}\right)\left(1-\frac{\cos^{2}\theta_{m}}{\cos^{2}\lambda_{0}}\right)}.$$

From Equation (19), using the variable change in Equation (41), we get:

(50) $${\left(\frac{d\theta }{dz}\right)}_{z=\pm d}=\frac{\cos\theta_{m}\sqrt{T(\theta_{m},\lambda_{0})}}{\sqrt{K_{1}+\left(K_{3}-K_{1}\right)\left(1-\frac{\cos^{2}\theta_{m}}{\cos^{2}\lambda_{0}}\right)}}\frac{\sin\lambda_{0}}{\cos\lambda_{0}},$$

where

(51) $$T\left(\theta_{m},\lambda_{0}\right)=\frac{\varepsilon_{0}\varepsilon_{a}U^{2}}{4d^{2}}+2A_{p}\left\{1-\xi_{p}\left[2-\cos^{2}\theta_{m}\left(1+\frac{1}{\cos^{2}\lambda_{0}}\right)\right]\right\}.$$

From Equations (49) and (50) in the assumptions of $\theta_{m}\to \pi /2$, we obtain:

(52) $$\sin\lambda_{0}=\frac{A_{S}\left(1+2\xi_{s}\right)}{\sqrt{K_{3}}\sqrt{\frac{\varepsilon_{0}\varepsilon_{\|}U_{S}^{2}}{4d^{2}}+2A_{p}\left(1-2\xi_{p}\right)}}$$

and, from Equations (48) and (52), we obtain for saturation voltage $\left(U_{s}\right)$:

(53) $$\tanh\left(\frac{d}{\sqrt{K_{3}}}\sqrt{\frac{\varepsilon_{0}\varepsilon_{\|}U_{S}^{2}}{4d^{2}}+2A_{p}\left(1-2\xi_{p}\right)}\right)=\frac{A_{S}\left(1+2\xi_{s}\right)}{\sqrt{K_{3}}\sqrt{\frac{\varepsilon_{0}\varepsilon_{\|}U_{S}^{2}}{4d^{2}}+2A_{p}\left(1-2\xi_{p}\right)}}.$$

Here, we can also obtain the corresponding values for the nematic sample where f=0, that is, $A_{p}=0$

(54) $$\tanh\left(\frac{d}{\sqrt{K_{3}}}\sqrt{\frac{\varepsilon_{0}\varepsilon_{\|}U_{S0}^{2}}{4d^{2}}}\right)=\frac{A_{S}\left(1+2\xi_{s}\right)}{\sqrt{K_{3}}\sqrt{\frac{\varepsilon_{0}\varepsilon_{\|}U_{S0}^{2}}{4d^{2}}}}.$$

This is also a transcendental equation that can be numerically solved by giving particular values to each parameter and by building data sets to be plotted.

## 3. Results and Discussions

**Figure 2:**
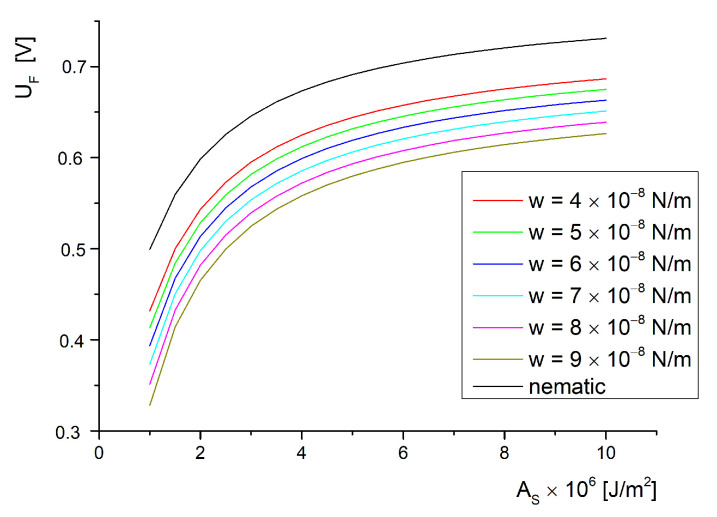
Freedericksz transition threshold versus anchoring strength energy for different interaction energies.

**Figure 3:**
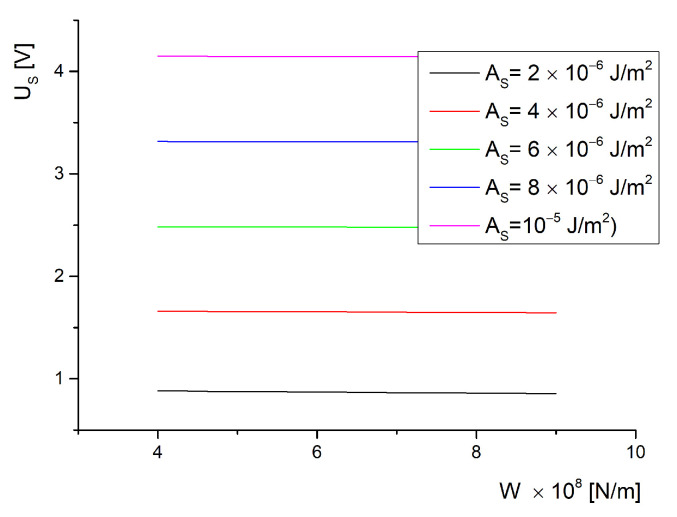
Saturation voltage versus interaction energy density between molecules and particles.

The Freedericksz transition threshold and the saturation field values were obtained by numerical solving of transcendental equations Equations (39) and (54). The values were obtained for certain values of anchoring energy on the substrate of the cell, $A_{S}$ in (Figure 2) and on interaction energy with the ferroparticle’s surface *w* in (Figure 3).

Considering an alternate voltage above 1 KHz applied to the LC-cell, we can neglect the polarisation effects and use an effective dielectric permittivity of the cell’s mixture ($\varepsilon_{a}$). The liquid crystal considered for simulation is 5CB for which we know $K_{1}=6.2\times 10^{-12}$ N, and $K_{3}=8.2\times 10^{-12}$ N. The composite contains a volumetric fraction of ferroelectric nanoparticles (f=0.005) with a cross-section diameter of $a=50\times 10^{-9}$ m, confined in a glass cell with a thickness of $d=10\times 10^{-6}$ m. The dielectric anisotropy of the sample is $\varepsilon_{a}=11.5$ şi and for the vacuum dielectric permittivity we have $\varepsilon_{0}=8.854\times 10^{-12}$ F/m. A planar anchoring of nematic molecules on the ferroparticle’s surface was assumed. The anchoring parameters used were $\xi_{S}=-0.2$ and $\xi_{p}=0.2$.

These parameters were replaced in Equation (39). A set of data was obtained by assuming values from $1\times 10^{-6}\;{J/m}^{2}$ to $10\times 10^{-6}\;{J/m}^{2}$ for the anchoring energy on the substrate of the cell ($A_{S}$) and a set of values from $4\times 10^{-8}\;N/m$ to $4\times 10^{-8}\;N/m$ for the anchoring energy on the ferroparticle’s surface (*W*). Thus, a table of data was created and the Freedericksz transition threshold $U_{F}$ was calculated for each set. The data were plotted in Figure 2. A similar procedure was used in Equation (40) for the nematic sample (without inserted nanoparticles) to obtain the “nematic” curve also presented in Figure 2.

As can be observed from Figure 2, the Freedericksz transition has an increase for low anchoring energies (below $4\times 10^{-6}$ J/m${}^{2}$) and tends to saturate above this value. So, for soft anchoring, there is a strong influence on the Freedericksz transition appearance while for strong anchoring, the field only affects the molecules in the middle of the cell, which are not affected by the surface constrains. The behavior is similar to that of the simple liquid crystal sample (presented in Figure 2 as nematic). In this case, the anchoring energy on the ferroparticle’s surface no longer exists so the values for this plot can be easily obtained for W=0. It can be noticed that the critical field decreases with the addition of nanoparticles, because the nematic curve is clearly above the others.

The nanoparticles’ insertion has a strong influence on the Freedericksz transition threshold. They are easily oriented by the applied field and orient the liquid crystal too. This aspect is implicitly described in Equation (9), where the interaction energy between particle and molecule is defined. Due to this interaction, the ferroparticle also affects the distortion angle and induces the transition earlier. We can say that the particles are pulling up the molecules on the field direction if the host has positive dielectric anisotropy and the molecular anchoring on the particle’s surface is almost planar. As a consequence, the ferroparticles orient the molecules at a lower voltage leading to a decrease in the energy used by the electric field to reorient the molecules, that is, a decrease of the Freedericksz transition threshold.

In [20] there is a review of ferroelectric nanoparticle dispersion in nematic liquid crystals, many of the cited references using the same mixture as the those for this study. Since the temperature is a key factor in critical field values’ determination as seen in [29], those values measured between 22 and 25 Celsius degrees are discussed. In [17,21], a decrease of about 20% in the Freedericksz transition threshold was observed when BaTiO_3_ particles were added at high frequencies of applied voltage (above 1 kHZ). These results are in good agreement with the plot given in Figure 2, where the curves for the ferroelectric particle composite are below the nematic curve. In [22], the effect is reversed, that is, there is an increase of the Freedericksz transition because the nanoparticle concentration is high (above 0.5 wt%) and they are gathering in microsized clusters. The same effect is observed in [23] at 1 wt% of BaTiO_3_.

**Figure 4:**
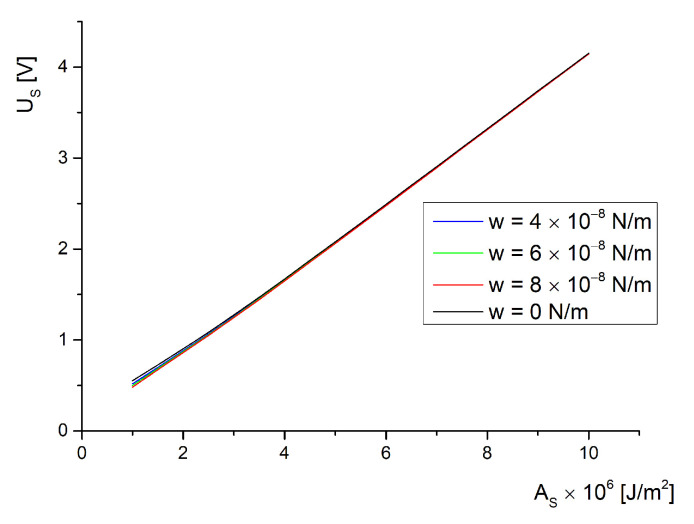
Saturation voltage versus anchoring energy of molecules on the substrate.

For a saturation critical field, a data set was obtained from the transcendental equation, Equation (54), which had the same range of values for the anchoring energies on the ferroparticle’s surface and on the substrate of the cell ($1\times 10^{-6}\;{J/m}^{2}$ to $10\times 10^{-6}\;{J/m}^{2}$ for $A_{S}$ and $4\times 10^{-8}\;N/m$ to $4\times 10^{-8}\;N/m$ for *W*). The data were plotted in Figure 3. As can be seen, there is a low influence of *W* on the saturation effect for every value of the anchoring strength on the substrate of the cell. This happens because if the field is strong enough to completely reorient the molecules, then the ferroelectric particles are already aligned with the field direction. Yet, when we use different values for the anchoring strength for each interaction’s energy, we obtain the plot given in Figure 4. Here, we notice a strong dependence of the saturation field on the anchoring strength because if a strong homogeneous alignment is provided by a certain coating, it will prevent the homeotropic alignment induced by the applied electric field. This effect is not influenced by the volumetric fraction of ferroparticles since the curves shown in Figure 4 are almost superposed.

**Figure 5:**
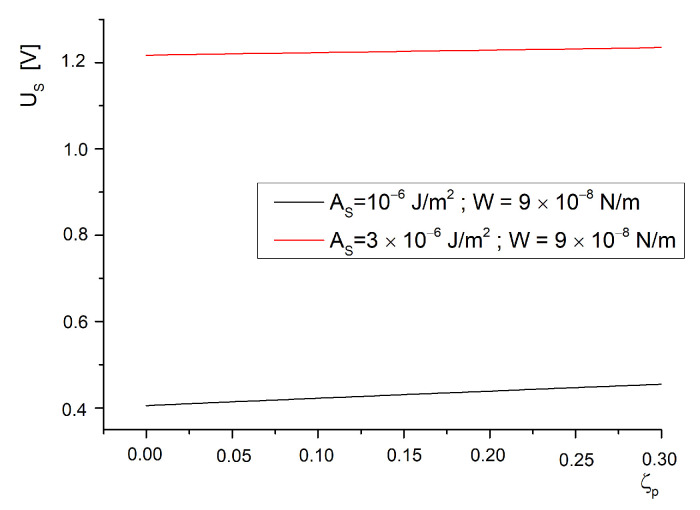
Saturation voltage versus anchoring anisotropy parameter for two different anchoring energies on the support surface.

Experiments presented in [23,24] confirm these results. The transition presented in [23] between the light and dark state of the liquid crystal indicates the values where the deviation angle reaches its maximum and the transition from planar orientation to almost homeotropic is achieved. In [23] the transition values are around 1 V for pure 5CB and present a slow decrease down to 0.85 V for the BaTiO_3_ containing sample. These results are in good agreement with the plots given in Figure 3 for low anchoring on the substrate of the cell. A different result is obtained for 1 wt% ferroparticle concentration because the particles are gathering together in microscopic bulks and the cylindrical model can no longer be applied. In [24], there is a low concentration of BaTiO_3_ used but the critical field has a strong decrease (about 70%). This can be explained by the oleic acid treatment that might have changed the molecular anchoring on the nanoparticle’s surface so the planar alignment considered in this model no longer applies. An interesting situation is the dependence of the saturation field on the anchoring energy presented in Figure 5, also from Equation (54). As can be observed, there is a small difference between the plots recorded for different interaction strengths with inserted nanoparticles, but this difference only occurs for soft anchoring on the cell support. For a rigid anchoring, the saturation field is the same with or without inserted particles.

## 4. Conclusions

A theoretical model is proposed to describe the influence of surface anchoring on the Freedericksz transition threshold and saturation field. This model can explain some of the physical interactions between liquid crystal molecules, cell substrate and ferroelectric nanoparticles. As shown in [20], there is much interest in these systems with a high potential for electro-optic applications. By choosing a certain preparation of ferroparticles (i.e., by coating or by fictionalisation), the electric Freedericksz transition can be considerably reduced without an increase in concentration, which could considerably affect the optical properties. The surface treatment of the substrate is also important because an equilibrium between good alignment and rapid reorientation is crucial for high performance devices.

## Sample Availability

Samples of the compounds are not available from the author.
